# Engineering Angular Dispersion via Hybrid Local–Nonlocal Metasurfaces

**DOI:** 10.1002/nap2.70169

**Published:** 2026-06-16

**Authors:** Shany Z. Cohen, Luna Sinitsky, Sukanta Nandi, Leeju Singh, Amos Sharoni, Tomer Lewi

**Affiliations:** ^1^ Faculty of Engineering Bar‐Ilan University Ramat Gan Israel; ^2^ Bar‐Ilan Institute for Nanotechnology and Advanced Materials Bar‐Ilan University Ramat Gan Israel; ^3^ Physics Department Bar‐Ilan University Ramat Gan Israel; ^4^ Indraprastha Institute of Information Technology, Delhi (IIIT‐Delhi) New Delhi India; ^5^ Center for Quantum Technologies Indraprastha Institute of Information Technology, Delhi (IIIT Delhi) New Delhi India

**Keywords:** angle‐limiting metasurfaces, angular filter, dielectric metasurfaces, non‐local metasurfaces, polarization‐insensitive, silicon photonics

## Abstract

Conventional dielectric metasurfaces typically exhibit broadband spectral and angular responses, as their individual elements inherently possess broadband characteristics with weak near‐field coupling between adjacent resonators. In contrast, nonlocal metasurfaces leverage collective electromagnetic interactions to achieve narrow spectral and angular selectivity, but often introduce polarization dependence arising from structural asymmetry. Here, we demonstrate a uniform and polarization‐insensitive metasurface that overcomes this typical trade‐off by deliberately combining local and nonlocal optical responses within a single platform. The proposed silicon nanopillar metasurface exhibits high transmission with pronounced angular discrimination through resonator engineering and periodicity‐driven control of collective modes, maintaining over 80% transmission within a ± 15° incidence cone while exhibiting strong attenuation outside this range. The engineered periodicity enables control over lattice‐mediated collective modes and substrate‐guided diffraction channels, with the latter becoming trapped within the substrate and thereby suppressing transmission into free space at larger incidence angles. Numerical simulations and experimental measurements show good agreement and validate the underlying design strategy. These findings establish a compact platform for angular‐selective optical components with potential applications in imaging, sensing, and LiDAR systems.

## Graphical abstract

A single‐layer hybrid local–nonlocal silicon metasurface is experimentally demonstrated as a polarization‐insensitive angular filter in transmission. By combining local nanopillar response, lattice‐mediated interactions near the Rayleigh anomaly, and diffraction‐assisted substrate coupling, the platform maintains high transmission within a ±15° cone while strongly suppressing larger‐angle light.

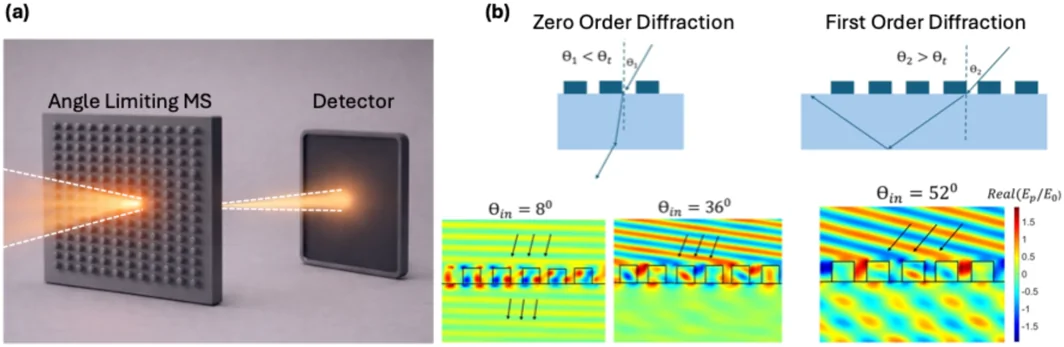

## 1 Introduction

Metasurfaces—ultrathin arrays of subwavelength optical resonators—have revolutionized flat optics by enabling unprecedented control over the phase, amplitude, polarization, and direction of light, in both passive [1, 2, 3, 4, 5, 6, 7, 8, 9, 10, 11] and actively tunable implementation [12, 13, 14, 15, 16, 17, 18, 19, 20, 21, 22, 23]. Among these, nonlocal metasurfaces have recently emerged as a distinct and powerful class, leveraging collective interactions across multiple unit cells to achieve functionality that extends beyond conventional, locally resonant designs [24, 25, 26, 27].

Unlike local metasurfaces, which operate primarily through isolated light–resonator interactions, nonlocal metasurfaces exploit in‐plane coupling, guided modes, and lattice resonances to manipulate light in the momentum space [24, 25, 26, 28]. This unique feature enables both angular and frequency selective responses, spatial frequency filtering, and wavefront shaping with resolution and tunability that are not attainable with traditional diffractive optics or local metasurfaces. Such capabilities have opened new avenues for phase spectroscopy [29, 30], beam shaping [26, 28, 29, 30, 31], angle multiplexing [32, 33, 34], angle‐selective absorption [35, 36, 37] and dynamic optical control [38, 39, 40]. In particular, angle‐selective metasurfaces have attracted growing interest for applications that require precise angular filtering, such as imaging, sensing and optical communications. In LiDAR systems, angle‐limiting components can suppress unwanted background light, enhance signal‐to‐noise ratios, and improve spatial resolution by restricting detection to specific angular ranges [41, 42, 43, 44].

A central mechanism underlying many nonlocal metasurface functionalities is the formation of collective lattice modes, in particular, the emergence of surface lattice resonances (SLRs) near the Rayleigh anomaly (RA). Although the RA marks the onset of a diffracted order, SLRs arise at slightly longer wavelengths, where the diffracted channel becomes evanescent. In this regime, the periodic lattice mediates long‐range interactions and couples the Rayleigh diffracted waves with the localized Mie resonances of the individual meta‐atoms. This hybridization reshapes the metasurface response, enabling excitation of higher‐order Mie modes [45], blue shifts of multipoles relative to isolated elements [46], and pronounced linewidth‐narrowing characteristic of SLRs [45, 46, 47, 48]. Coherent interference between SLRs and Mie multipoles can further give rise to directional scattering effects such as the resonant lattice Kerker condition [45, 49, 50]. Importantly, SLRs are highly tunable through the array periodicity, incidence angle, and surrounding refractive index, enabling precise engineering of angle‐dependent responses in nonlocal metasurfaces [50, 51, 52, 53].

Despite this progress, achieving well‐defined angular control in compact planar optical platforms remains challenging for integrated photonic systems [1, 29, 32, 36, 54, 55]. Many existing metasurfaces exhibit uncontrolled angular dispersion, operate only over narrow incidence windows, or rely on polarization selective responses, which limits their practical deployment in realistic applications [29, 32, 36, 54, 55]. These limitations motivate the development of architectures that merge local and nonlocal design principles, such that the strong unit‐cell level control provided by local resonances can be combined with the collective, angle‐sensitive response enabled by lattice‐mediated interactions. Recent studies have shown that such local–nonlocal combinations can unlock functionality beyond those of purely local metasurfaces, including enhanced spectral selectivity, controlled thermal emission, wavefront manipulation and compact flat‐optics implementation [56, 57]. However, in these representative studies, the combined functionality was typically realized using bilayer metasurfaces or systems composed of two distinct metasurfaces, rather than being jointly engineered within a single patterned layer. In addition, these works were primarily theoretical or design‐oriented, and an experimental demonstration of a single‐layer transmissive local–nonlocal metasurface for angle‐limiting operation has remained lacking. Therefore, translating this hybrid design philosophy into a compact, single‐layer transmissive metasurface that simultaneously provides a broad angular passband and polarization insensitivity, along with experimental validation operation remains nontrivial.

In this work, we theoretically design and experimentally demonstrate a single‐layer local–nonlocal metasurface that addresses several of the above limitations. Specifically, we present a dielectric transmission‐mode metasurface operating around a central wavelength of 940 nm, in which local resonator effects and lattice‐mediated nonlocal interactions are jointly engineered to realize angle‐limiting behavior. The metasurface exhibits high transmission (> 80%) within a relatively broad angular window (± 15°), while effectively suppressing off‐axis light beyond this range. An additional advantage of the design is its polarization‐insensitive response, which distinguishes it from many phase‐gradient metasurfaces and meta‐gratings that often exhibit strong polarization sensitivity [29, 34, 55]. The resulting angular filtering behavior, arising from the interplay of local and nonlocal characteristics of the structure, is confirmed by both numerical simulations and experimental measurements, showing good agreement.

## 2 Results and Discussion

### 2.1 Design and Optimization

Figure 1 illustrates the operating principle of the angle‐limiting metasurface. Figure 1a presents a conceptual schematic of the device functionality, where a broad angular field of view is incident on the metasurface, whereas only a narrower angular range is transmitted toward the detector, highlighting the angular filtering behavior. Figure 1b summarizes the three operating regimes of the device, which are discussed in detail later. At low incidence angles (|${ϴ}_{\text{in}}$| < 15°), transmission is dominated by the off‐resonant local response of the individual nanopillars, and only the zeroth diffraction order is supported yielding high transmission. At intermediate angles (15° < |${ϴ}_{\text{in}}$| < 44°), the response becomes increasingly influenced by lattice‐mediated interactions as the system approaches the RA, resulting in decreased forward transmission. The corresponding field profile at ${ϴ}_{\text{in}}=36{}^{\circ}$ indicates strong interaction of the incident field with the nanopillar array, consistent with enhanced nonlocal lattice‐mediated coupling in the SLR‐like regime [45, 46, 47, 48]. In this regime, coherent lattice resonances can redistribute scattering into reflected and other radiative channels [45, 49, 50] suggesting that the reduced forward transmission arises primarily from radiative redistribution rather than from material absorption, which is negligible in the present Si/SiO_2_ platform around 940 nm. At larger angles (|${ϴ}_{\text{in}}$| > 44°), once the first diffraction order becomes accessible inside the substrate, the periodic lattice provides the additional in‐plane momentum required to couple incident light into this channel. Because this diffracted light propagates in the substrate at an angle exceeding the critical angle at the quartz‐air interface, it remains confined within the substrate, resulting in strong suppression of forward transmission into air. Importantly, the key contribution of the present work lies not in high‐angle attenuation by itself, but in the experimental realization of a single‐layer transmissive hybrid local–nonlocal metasurface whose angular response is deliberately shaped through the interplay of local nanopillar response, lattice‐mediated collective effects, and diffraction‐assisted substrate coupling. This enables a compact platform in which the angular passband, diffraction threshold, and on‐axis transmission can be jointly engineered while preserving near polarization‐insensitive operation.

**FIGURE 1:**
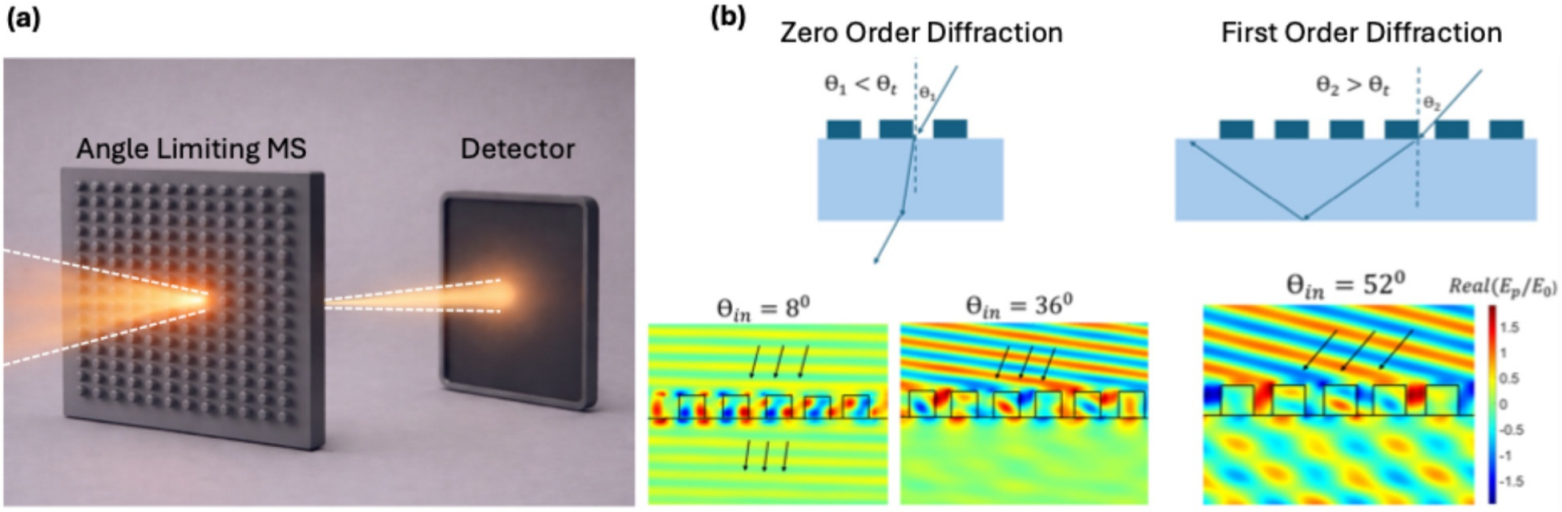
(a) Schematic illustration of the angle‐limiting response of the metasurface, showing a broad angular field of view at the input and a narrower transmitted angular range at the output. (b) Cross‐sectional schematic and simulated field profiles for three representative incidence angles, illustrating the operating principle of the device. The field profiles show the real part of the normalized electric field component $E_{p}/E_{0}$ for p‐polarized illumination. At low incidence angles ($\theta_{\text{in}}=8{}^{\circ}$), transmission is dominated by the off‐resonant local response of the nanopillars, and only the zeroth diffraction order is present. At intermediate angles ($\theta_{\text{in}}=36{}^{\circ}$), the response is increasingly influenced by lattice‐mediated interactions as the system approaches the RA. The corresponding field profile indicates strong interaction of the incident field with the nanopillar array, consistent with enhanced nonlocal lattice‐mediated coupling and reduced forward transmission, likely accompanied by redistribution into reflected and other radiative lattice‐mediated channels. At higher angles ($\theta_{\text{in}}=52{}^{\circ}$), the first diffraction order becomes accessible inside the substrate, enabling coupling of incident light into a substrate‐side diffractive channel. Since this light propagates beyond the critical angle at the quartz–air interface, it remains trapped within the substrate and forward transmission into air is strongly suppressed. The field profiles shown for incident angle of ${ϴ}_{\text{in}}$ = 52° confirm this behavior by demonstrating that the energy becomes confined within the substrate rather than radiating into free space.

The metasurface design comprises an array of silicon nanopillars arranged in periodic structure on top of a quartz substrate, with geometry optimized to achieve high transmission at a central wavelength of $\lambda_{0}=940$ nm and to provide an angular‐limiting cone of ± 15°. Both parameters were selected to correspond to typical operating conditions of near‐infrared laser‐based 3D imaging systems, although the design concept is general and can be adapted to other wavelengths and angular ranges for diverse optical applications.

In many metasurface designs, there tends to be a trade‐off between resonance quality (Q) factor and angular tolerance. High‐Q metasurfaces, which exhibit narrow spectral linewidths due to low radiative (and material) losses, are often angle‐sensitive. Their optical response is sharply tuned to specific in‐plane wavevectors, meaning even slight deviations in the angle of incidence can shift the system off‐resonance, leading to significant changes in transmission or reflection. This angular selectivity is often exploited in momentum‐domain metasurfaces for applications such as angular filtering and spatial frequency discrimination. In contrast, low‐Q metasurfaces, whether due to deliberately broadened resonances or local nonresonant responses, tend to support a much wider angular bandwidth. Their performance remains relatively stable over a broad range of incidence angles, making them more robust for applications requiring angle tolerance. Our design leverages this trade‐off by achieving effective angular filtering within a controlled window, balancing angle selectivity with high transmission.

We selected silicon as the resonator material because of its mature fabrication process and its high refractive index (*n*∼3.6) enables strong field confinement and compact Mie‐type resonances, which is advantageous for realizing the targeted hybrid local–nonlocal response in a thin transmission platform. Although low‐loss platforms such as TiO_2_ and Si_3_N_4_ are good alternatives, the present Si/SiO_2_ device benefits from higher index contrast and operates at 940 nm where material absorption of silicon is negligible [58, 59].

We employed finite‐difference time‐domain (FDTD) simulations to characterize and optimize the spectral performance of our metasurface. The design was first optimized by sweeping through different values of resonator radius and height, while monitoring the transmission at normal incidence for *λ*_0_ = 940 nm, as shown in Figure 2a. The white circle in the transmission map marks the geometry with radius *r* = 145 nm, height *h* = 370 nm, and lattice period *Λ* = 440 nm, which provides high transmission at *λ*_0_ with a resonance FWHM ≈ 150 nm. This moderate spectral bandwidth is consistent with our design strategy: it is broad enough to support angular tolerance while still providing spectral selectivity that enables suppression of off‐axis transmission.

**FIGURE 2:**
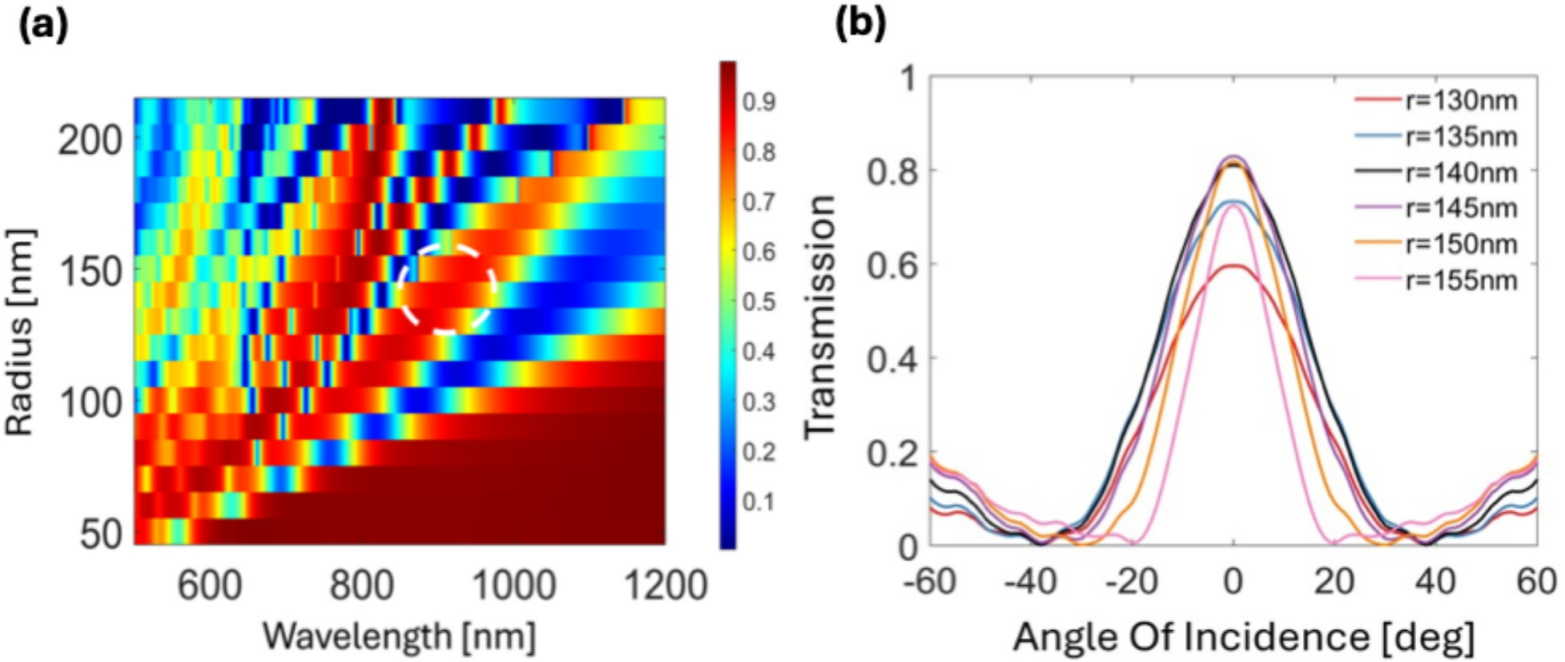
Design and optimization of single resonator dimensions. (a) Transmission map showing the dependence of pillar radius at normal incidence (for fixed height *h* = 370 nm). The white circle indicates the selected geometry (*r* = 145 nm, *h* = 370 nm, *Λ* = 440 nm), yielding high transmission at *λ*_0_ = 940 nm with a spectral bandwidth of ∼150 nm; (b) angle‐resolved transmission for selected radii, illustrating how the resonator size influences the angular response.

After identifying the resonator radius regime that yields high on‐axis transmission, we further refined the nanopillar size by analyzing the transmission characteristics as a function of incidence angle for different nanopillar radii, as shown in Figure 2b. This step is essential because the angular selectivity of the metasurface arises from the interplay between the intrinsic resonances of the individual pillars (local response) and the angle‐dependent coupling to lattice‐mediated modes (nonlocal response).

Figure 2b shows that even small variations in the pillar radius produce significant changes in the angular response, including reduced transmission and narrowing or broadening of the angular passband. These results highlight the strong sensitivity of the design to the resonator geometry and, at the same time, emphasize the fabrication challenges associated with maintaining such dimensional precision. Based on these results, the resonator with *r* = 140 nm provides the desired angular transmission window of ± 15°.

In addition to tuning the nanopillar dimensions, the angular behavior is strongly influenced by the periodicity of the array and the RA. The RA occurs when a diffracted order becomes grazing in a given medium, that is :

(1) $$|k_{∥}+G_{mn}|=n_{\text{med}}k_{0},$$

where $k_{∥}$ is the in‐plane component of the incident wavevector, $G_{mn}$ is the reciprocal‐lattice vector of the (*m*, *n*) diffraction order, $n_{\text{med}}$ is the refractive index of the medium in which the order is considered, and $k_{0}=2\pi /\lambda$. For incidence in the x–z plane on a square lattice of period Λ, this becomes:

(2) $${\left(n_{\text{inc}}k_{0}\;\sin\;\theta +\frac{2\pi m}{\Lambda }\right)}^{2}+{\left(\frac{2\pi n}{\Lambda }\right)}^{2}={\left(n_{\text{med}}k_{0}\right)}^{2}.$$

At normal incidence, this reduces to $\lambda_{\text{RA}}^{\left(m,n\right)}=\frac{n_{\text{med}}\Lambda }{\sqrt{m^{2}+n^{2}\;}}$.

In our case, the lattice period must satisfy two complementary criteria. First, the metasurface should operate slightly on the long‐wavelength side of the substrate RA, $\lambda >\lambda_{\text{RA}}^{\left(m,n\right)}$, where $n_{\text{med}}=n_{\text{sub}}$, such that collective lattice interactions become significant [45, 46, 47, 48]. These nonlocal effects help enforce a uniform transverse response across the metasurface and contribute to enhanced control over the angular dependence. Second, to achieve efficient suppression of transmission at large incidence angles, the periodicity is chosen such that the first diffracted order becomes accessible at those angles. Once this channel opens, the incident light is redirected into higher‐angle propagation within the quartz substrate, where it subsequently encounters the quartz–air interface at angles exceeding the critical angle; and is therefore trapped by total internal reflection. This dual role of the periodicity, promoting nonlocal lattice‐mediated interactions and SLR‐like effects near the RA, and redirecting diffraction channels into substrate guided modes at higher angles, forms the basis of our angle‐selective design.

Figure 3a presents the periodicity‐optimization process and highlights how the lattice period serves as an additional degree of freedom for tailoring the nonlocal response. The RA is indicated by the black dashed line, whereas the resonances of the structure appear as dips in the transmission spectrum (dark colors). In the vicinity of the RA, on the long wavelength side, SLR‐like behavior is observed as a red shift of the resonances at λ≅850 nm with increasing periodicity, reflecting enhanced hybridization between localized meta‐atom resonances and diffractive lattice modes. Further increase of the periodicity leads to the emergence of a narrow, high‐Q resonance pinned close to the RA, which is consistent with the characteristic fingerprint of SLRs. In the final design, a period of *Λ* = 440 nm (white dashed line) was chosen such that the first diffracted order emerges at an incidence angle of *θ*_*t*_ ≈ 44°. For ${ϴ}_{\text{in}}$ > *θ*_*t*_, the incoming radiation couples efficiently to the (−1, 0) diffracted order, which becomes propagating in the substrate and is redirected into substrate‐guided modes, thereby suppressing transmission into air. The resulting simulated angle‐resolved transmission map (Figure 3b) exhibits the targeted behavior: high transmission (> 80%) for |${ϴ}_{\text{in}}$| < 15°, and strong attenuation at larger angles. We note that the transmission in Figure 3b is calculated inside the substrate. Therefore, the elevated intensity appearing beyond the RA represents guided or confined energy within the substrate, rather than propagating power in free space.

**FIGURE 3:**
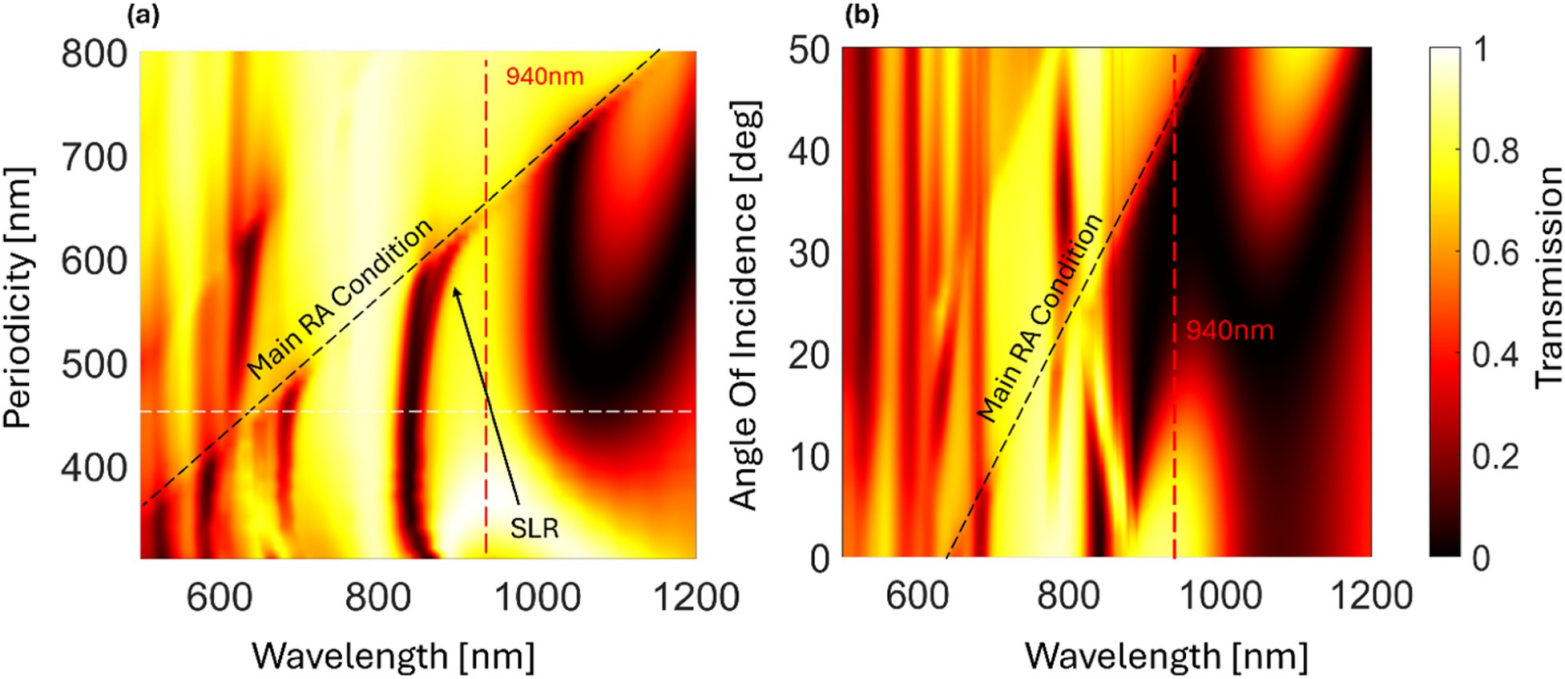
Effect of lattice periodicity. (a) Transmission map as a function of wavelength and periodicity. SLR‐like behavior is observed indicated by the red‐shift, together with the emergence of a sharp resonance close to RA at λ≅850 nm. The dashed white line marks the selected design at *Λ* = 440 nm, chosen such that the first diffracted order appears at *θ*_t_ ≈ 44°. (b) Angle‐resolved transmission map illustrating high transmission for |*θ*| < 15°, reduced transmission for intermediate angles (15°–44°) due to increasing lattice‐mediated (SLR‐like) interactions near the RA, and strong attenuation for |*θ*| > 44° due to coupling into the first diffracted order and subsequent confinement of energy within the substrate.

The optimization of nanopillar size and lattice periodicity is inherently codependent. Adjusting the resonator dimensions influences the spectral width and local phase response, while modifying the periodicity tunes the onset of nonlocal effects and the diffraction threshold. These two factors were therefore iteratively optimized to achieve the desired combination of high transmission for low angles and robust suppression at larger angles.

Following the iterative optimization of the nanopillar geometry and lattice periodicity, Figure 4a presents the simulated transmission spectrum and transmission phase of the optimized structure (h=370 nm, r=140 nm, Λ=440 nm), demonstrating high transmission at the target wavelength $\lambda_{0}=940$ nm. The phase response indicates that the metasurface operates in an off‐resonant regime around 940 nm at normal incidence. Figure 4b further shows that the optimized design is nearly polarization‐insensitive. The angular responses under s‐polarized and p‐polarized illumination are nearly identical over the transmission window of interest. A small difference becomes visible only at larger incidence angles, above approximately 50°, near and beyond the onset of the first substrate‐side diffractive order. This suggests that the weak s‐/p‐polarized splitting arises from slightly different polarization‐dependent coupling efficiencies into the newly accessible diffractive channel.

**FIGURE 4:**
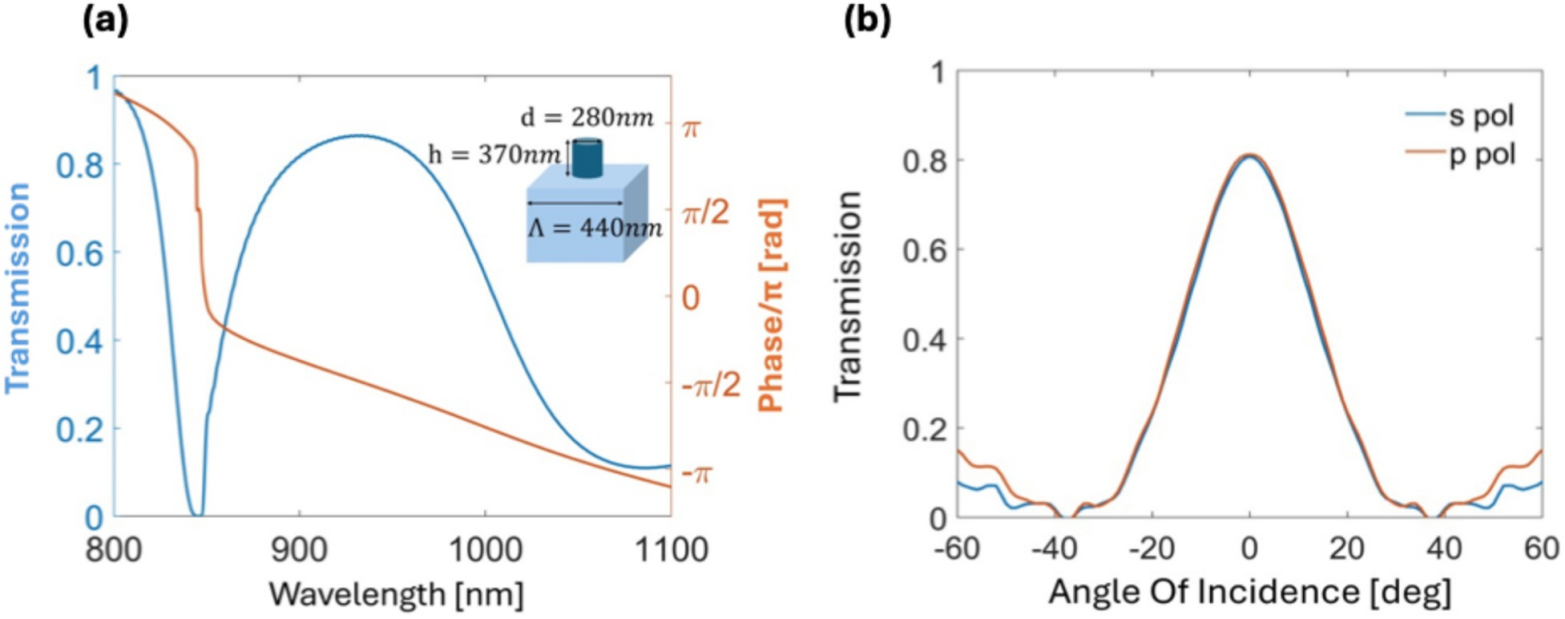
Response of the optimized structure. (a) Simulated transmission spectrum and transmission phase of the optimized structure under normal incidence. The inset shows the corresponding unit‐cell geometry. (b) Simulated angular transmission under s‐polarized and p‐polarized illumination, showing nearly identical responses over the transmission window of interest. A small difference becomes visible only above approximately 50°, near and beyond the onset of the first substrate‐side diffractive order, where weak s‐/p‐polarized splitting is attributed to slightly different polarization‐dependent coupling efficiencies into the newly accessible diffractive channel.

### 2.2 Device Fabrication and Experimental Results

The angle‐limiting nanopillar Si metasurface was fabricated on a double side polished 500 μm thick quartz substrate. The quartz substrate was cleaned using standard solvent and oxygen plasma treatments. A 370 nm‐thick undoped silicon film was first deposited on the quartz substrate by electron‐beam evaporation. The metasurface pattern was then defined by electron‐beam lithography using an MMA/PMMA bilayer resist, followed by Cr deposition and lift‐off to form a hard mask. This Cr hard mask was subsequently used to transfer the pattern into the silicon layer by dry reactive‐ion etching (SF_6_/C_4_F_8_) yielding well‐defined silicon nanopillars with controlled dimensions. A scheme of the full fabrication process is described in Figure 5a. A scanning electron microscope (SEM) image of the metasurface is presented in Figure 5b. The image confirms that the pillar dimensions and periodicity are within ± 5 nm fabrication tolerance. We note that some nonuniformity in the pillar etch depth is visible in the fabricated structure, which may lead to small deviations in the angular response relative to the idealized unit‐cell prediction.

**FIGURE 5:**
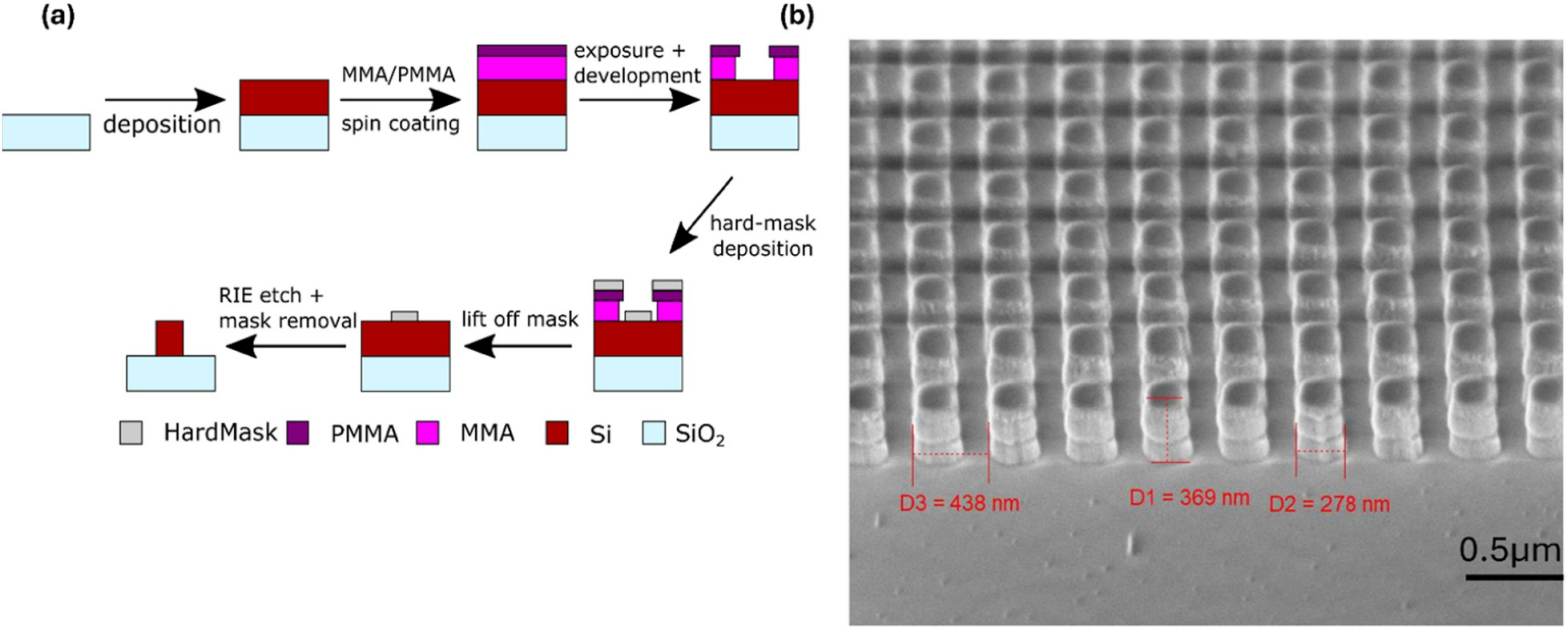
(a) Fabrication process scheme. (b) SEM image of the angle limiting metasurface, after etching and mask removal.

To characterize the scattering properties of the angle‐limiting metasurface, we used the optical setup shown in Figure 6a. A diode laser (LDC205C, Thorlabs) emitting at 940 nm with maximum output power 200 mW was used as the light source. The beam was collimated and then focused onto the sample, which was mounted on a rotating stage (PRMTZ8 Rotation Stage, Thorlabs). For each angle of incidence, the transmitted light was directed either to a CMOS camera for imaging or to a power meter for quantitative transmission measurements.

**FIGURE 6:**
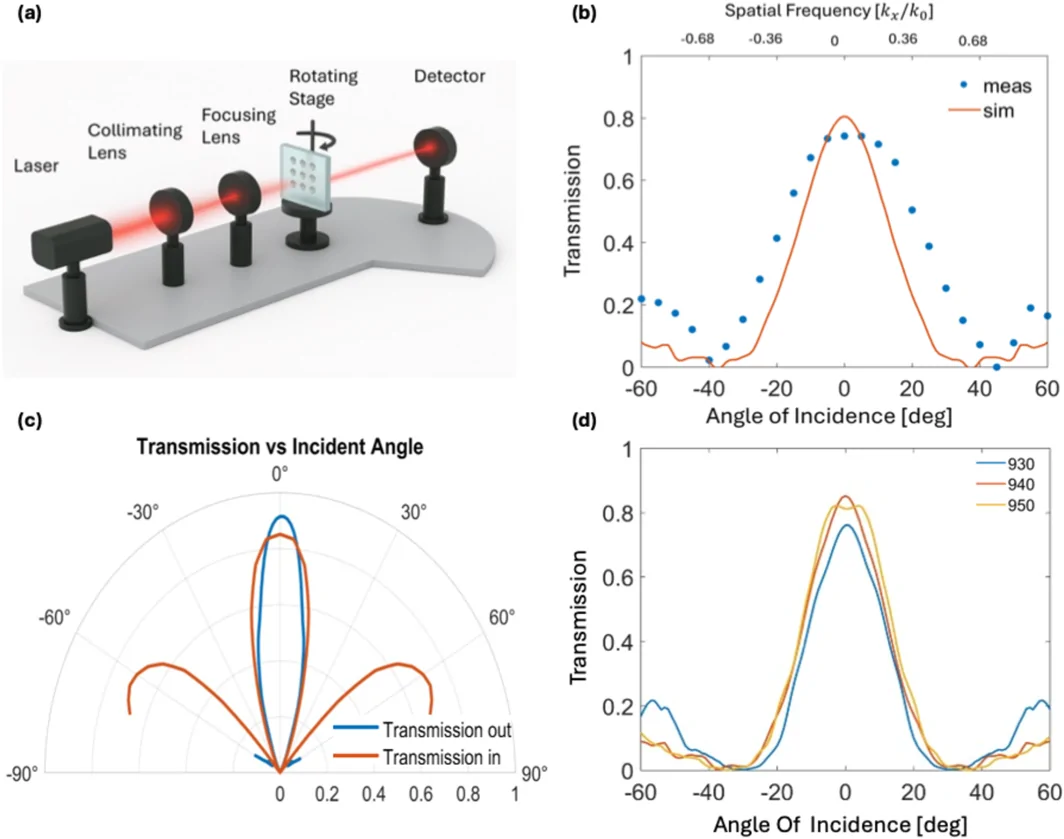
Angle‐resolved characterization of the metasurface performance. (a) Schematic of the optical measurement setup used for angle‐resolved transmission characterization; (b) measured (blue dots) and simulated (orange curve) transmission intensity as a function of incidence angle, showing high transmission within the low‐angle regime ${ϴ}_{\text{in}}<\pm \;15{}^{\circ}$ and strong attenuation outside this range. (c) Polar plot of simulated transmission intensity versus angle of incidence. The orange curve represents the transmission intensity inside the quartz substrate, showing enhanced transmission for 45° < *θ*_in_ < 70° due to excitation of the first diffraction order, whereas the blue curve depicts the transmitted intensity into air, which is strongly suppressed as a result of confinement within the substrate. (d) Simulated transmission intensity versus angle of incidence for wavelengths *λ* = 940 ± 10 nm. The metasurface maintains robust angular performance across the spectral range, with optimal transmission at the central wavelength (*λ*_0_ = 940 nm).

The measured angular response (spatial frequency) of the metasurface is presented in Figure 6b, demonstrating its angular filtering capabilities. High transmission is achieved for small angles of incidence (${ϴ}_{\text{in}}$ < ± 15°), whereas transmission is significantly attenuated outside this angular range. Figure 6b also compares the simulated and measured angular transmission profiles, under p‐polarized excitation, showing good agreement between simulations (orange curve) and experimental measurements (blue dots). The measured transmission peak is slightly reduced and the angular response is broader than predicted by simulations. This discrepancy can be attributed to fabrication‐induced imperfections, including minor deviations in nanopillar radius (see Figure 5b), sidewall roughness, and nonuniform etch depth, none of which are included in the simulations. In addition, the experimentally observed high‐angle attenuation ($\theta_{\text{in}}>45{}^{\circ}$) is weaker than predicted numerically. Beyond fabrication imperfections, this difference may also be influenced by the finite sample size, finite beam divergence, and residual edge leakage in the experiment. Nevertheless, the measured device retains the intended angle‐limiting behavior, indicating that the underlying effect is robust to the fabrication imperfections present in the current sample. Tolerance‐aware optimization and etch‐bias compensation therefore represent natural next steps for further improving device performance and increasing robustness to fabrication‐induced deviations.

To validate the attenuation mechanism at large angles, Figure 6c shows a polar plot of the simulated transmission intensity, displaying both the power coupled into the substrate and the power transmitted into air as functions of incidence angle. For angles greater than 44°, the transmitted intensity within the substrate (orange curve) increases sharply, whereas the total transmission into air (blue curve) remains low. This behavior confirms that beyond the threshold angle (*θ* > *θ*_t_ ≈ 44°), the first diffraction order is efficiently excited, and light becomes confined within the substrate. This condition corresponds to the regime where the propagation angle of the first diffracted order exceeds the critical angle of the quartz–air interface, resulting in total internal reflection and strong suppression of outward transmission.

To demonstrate that the dominant diffraction order excited at large incidence angles is the first one (−1, 0), we performed coupling‐efficiency calculations defined as:

$$T\left(n,m\right)=\frac{\text{Transmitted}\;\text{power}\;\text{to}\;\left(n,m\right)\;\text{order}}{\text{Total}\;\text{transmitted}\;\text{power}}.$$

For an incidence angle of *θ*_in_ = 56° and wavelength *λ*_0_ = 940 nm, the transmitted intensity within the substrate is distributed between the (0, 0) and (−1, 0) diffraction orders as follows:

T(0,0)=0.071,T(−1,0)=0.929.

These results confirm that the dominant transmission pathway indeed corresponds to the first diffracted order, consistent with the strong attenuation of forward transmission observed experimentally and in simulations.

Finally, we evaluated the robustness of the angular filtering effect across a narrow spectral window. Figure 6d shows the angular response for wavelengths ± 10 nm around the central design wavelength (*λ*_0_ = 940 nm). All three curves display nearly identical angular dependence, with maximum transmission for *θ*_in_ < 15°, verifying the effectiveness and spectral stability of the optimized design.

## 3 Conclusions

In summary, the proposed metasurface demonstrates efficient angular filtering, combining high transmission within a well‐defined angular cone with strong attenuation at larger incidence angles. This response originates from the interplay between the intrinsic resonances of the individual metaatoms at small incidence angles, enhanced nonlocal lattice‐mediated (SLR‐like) interactions in the vicinity of the RA at intermediate angles, and, at larger angles, efficient coupling to the first diffracted order, which redirects the incident energy into substrate‐guided modes and thereby suppresses transmission into air. Experimental measurements show good agreement with numerical simulations, validating the underlying design approach. Although the present work is focused on the realization and characterization of the angle‐limiting metasurface itself, the demonstrated angular selectivity, together with the ability to engineer the angular passband and diffraction threshold through the metasurface geometry, suggests potential relevance for future integration into imaging, sensing, and LiDAR systems, where directional filtering and background suppression are important. Overall, these results establish a promising route toward compact angular metafilters with tunable wavelength and angular characteristics enabled by engineered nonlocal dispersion.

## Author Contributions

**Shany Z. Cohen:** investigation, data curation, methodology, validation, visualization, writing – original draft, software, formal analysis, writing – review and editing. **Luna Sinitsky:** investigation, validation, visualization, methodology, writing – original draft, data curation. **Sukanta Nandi:** investigation, writing – original draft, formal analysis, methodology. **Leeju Singh:** investigation. **Amos Sharoni:** investigation, supervision, writing – review and editing. **Tomer Lewi:** conceptualization, investigation, funding acquisition, writing – review and editing, project administration, supervision, formal analysis, methodology, visualization.

## Conflicts of Interest

The authors declare no conflicts of interest.

## Data Availability

The data that support the findings of this study are available from the corresponding author upon reasonable request.
